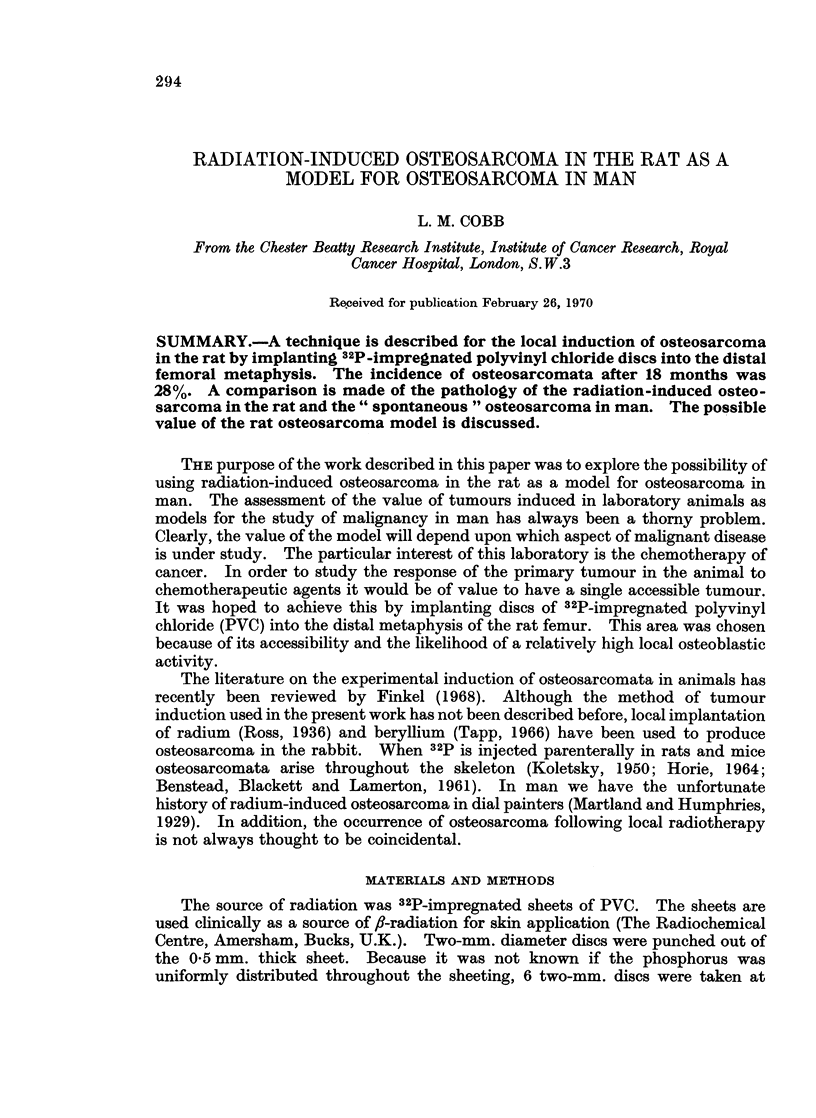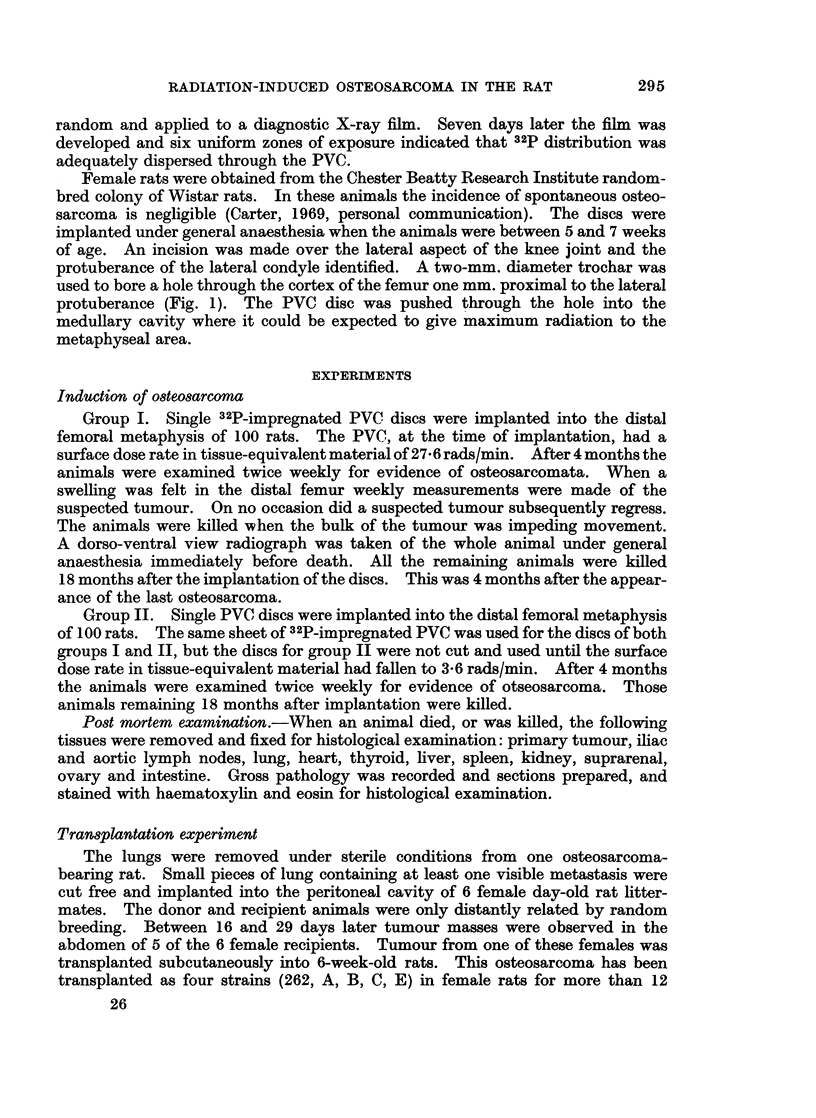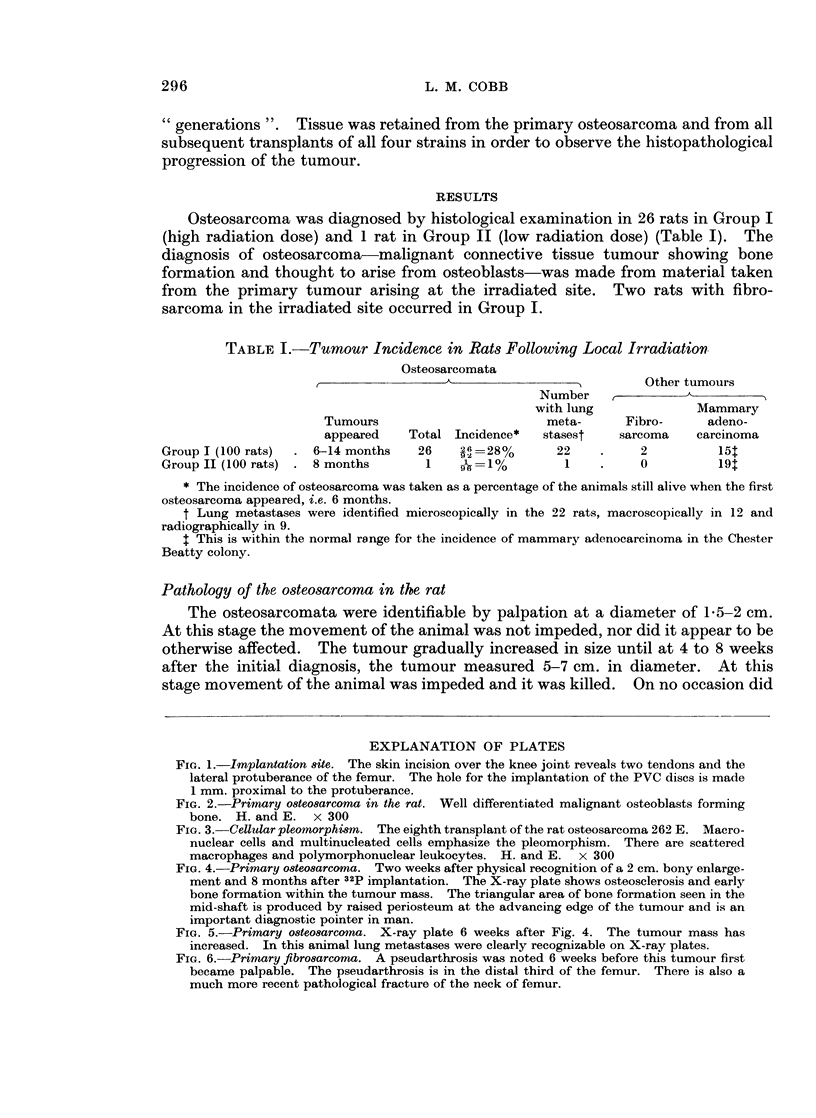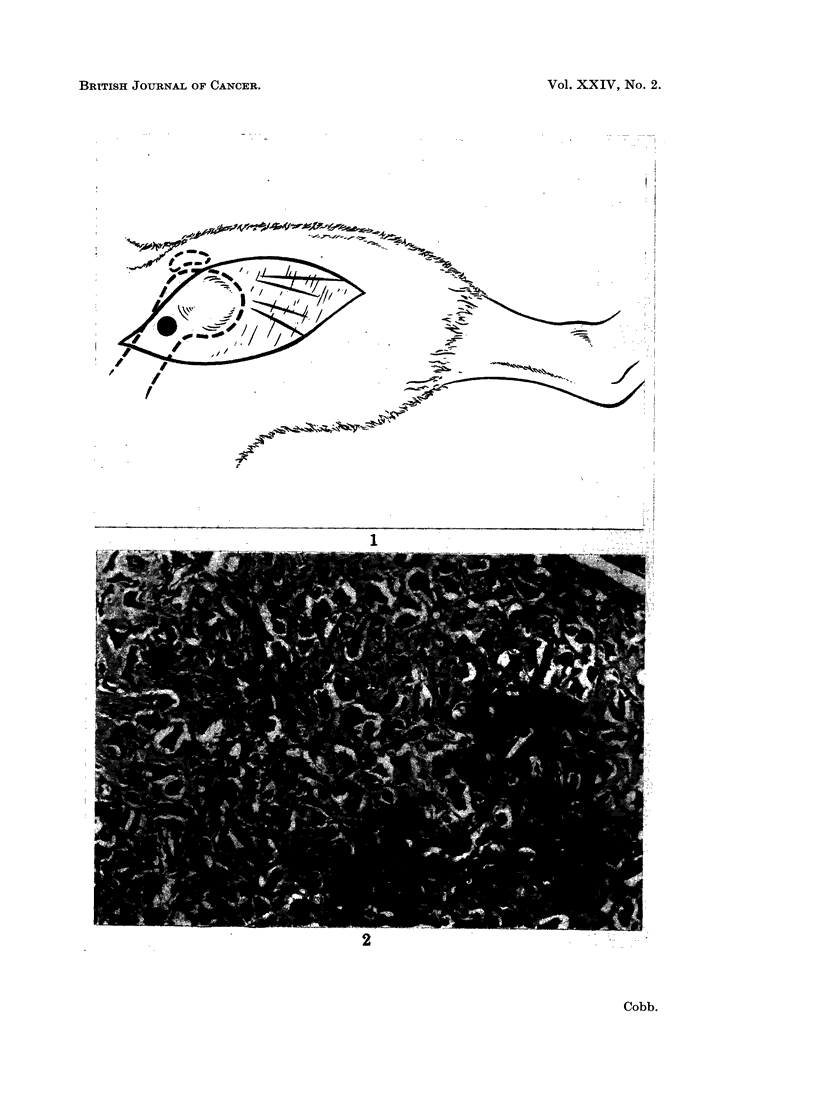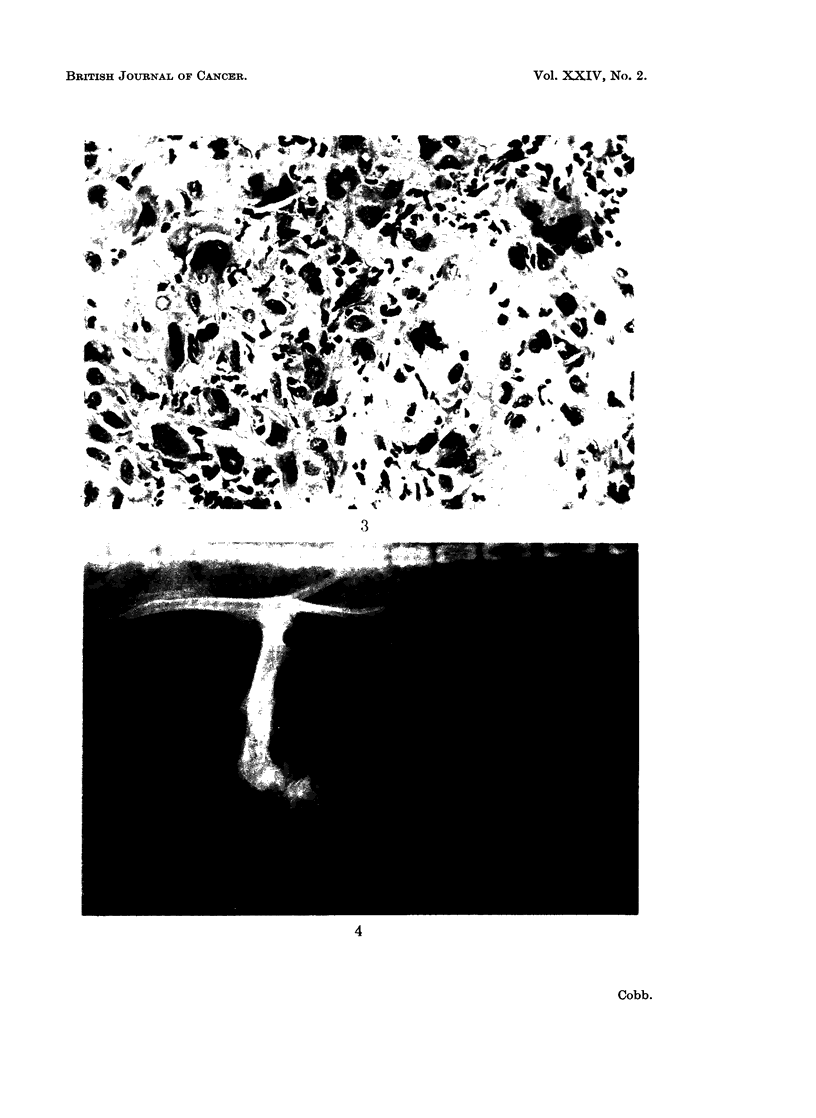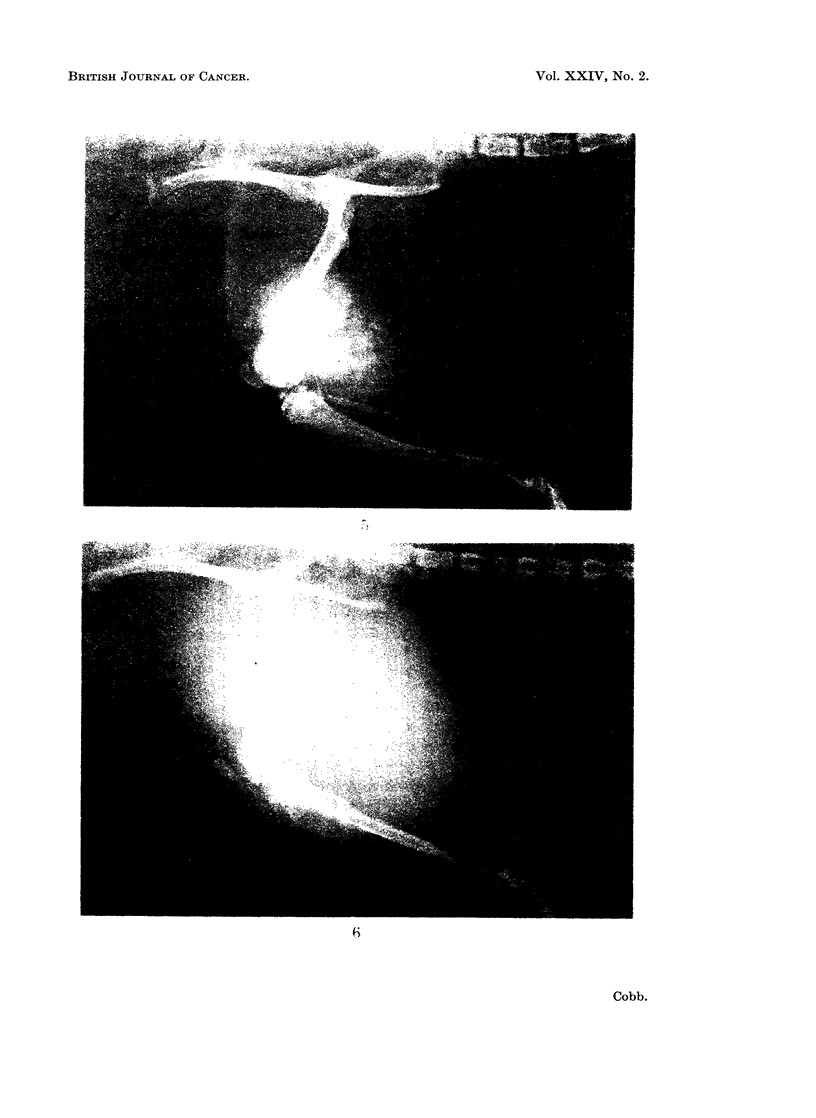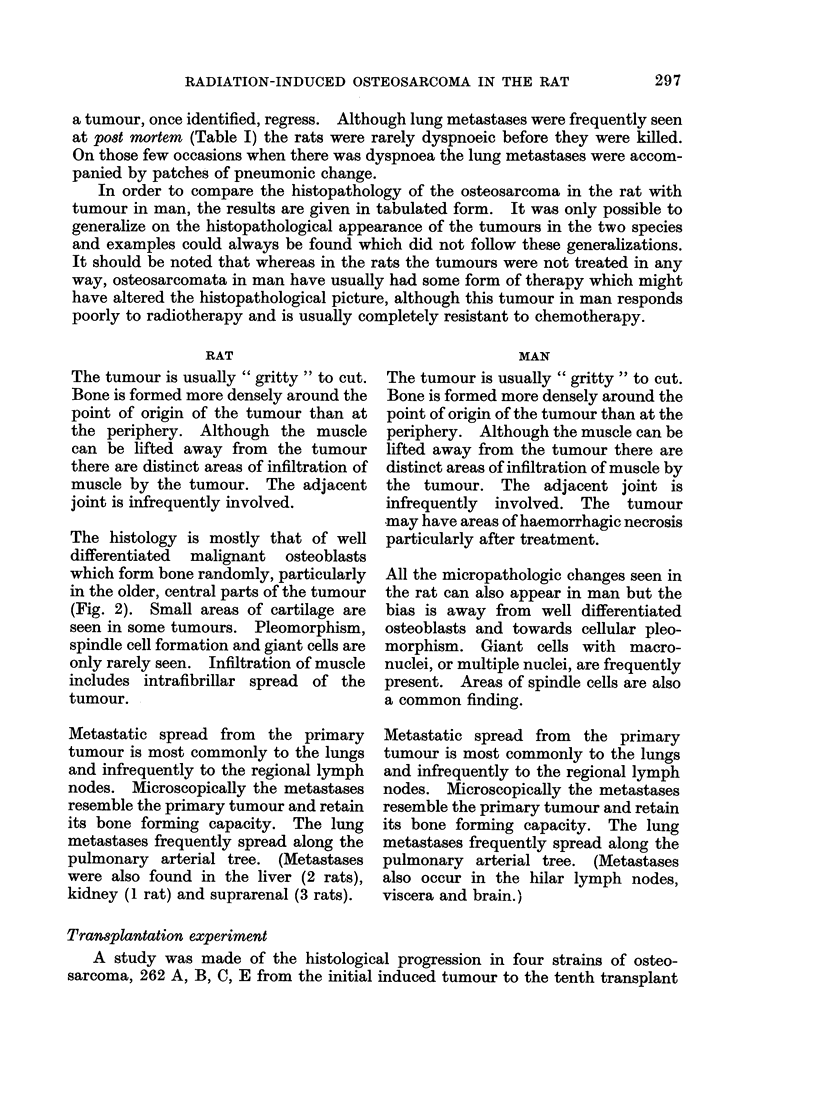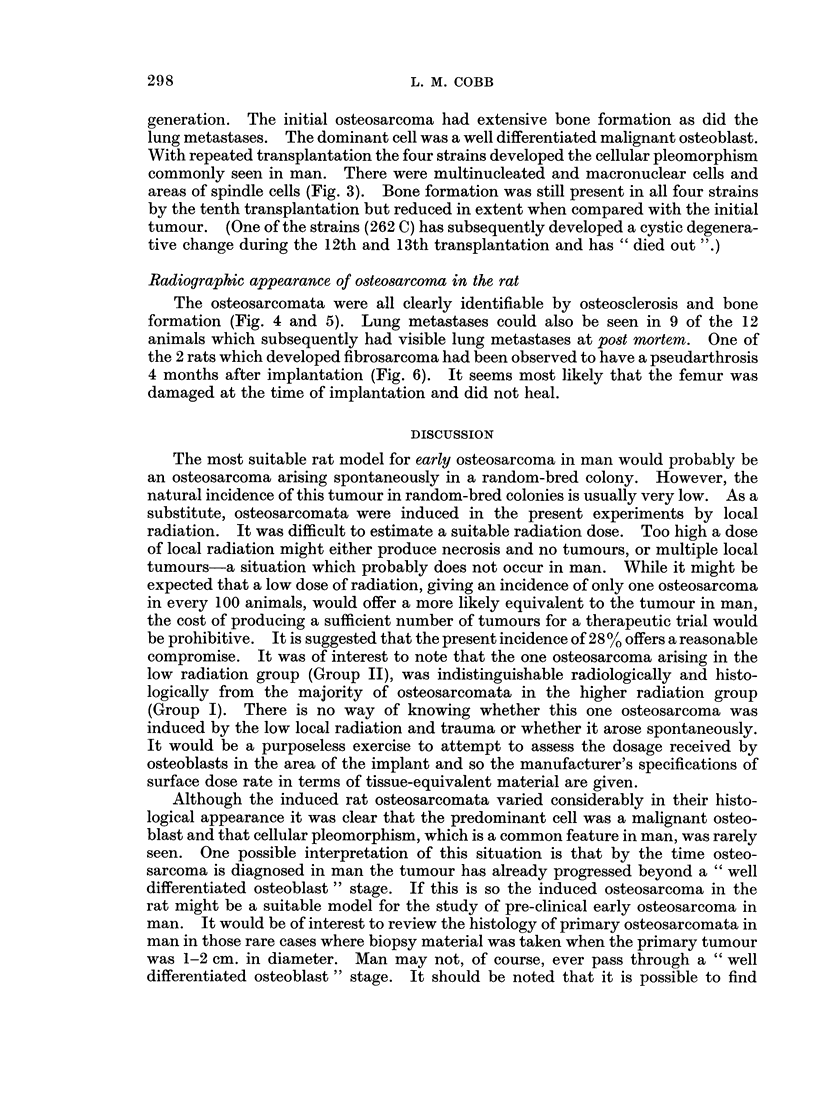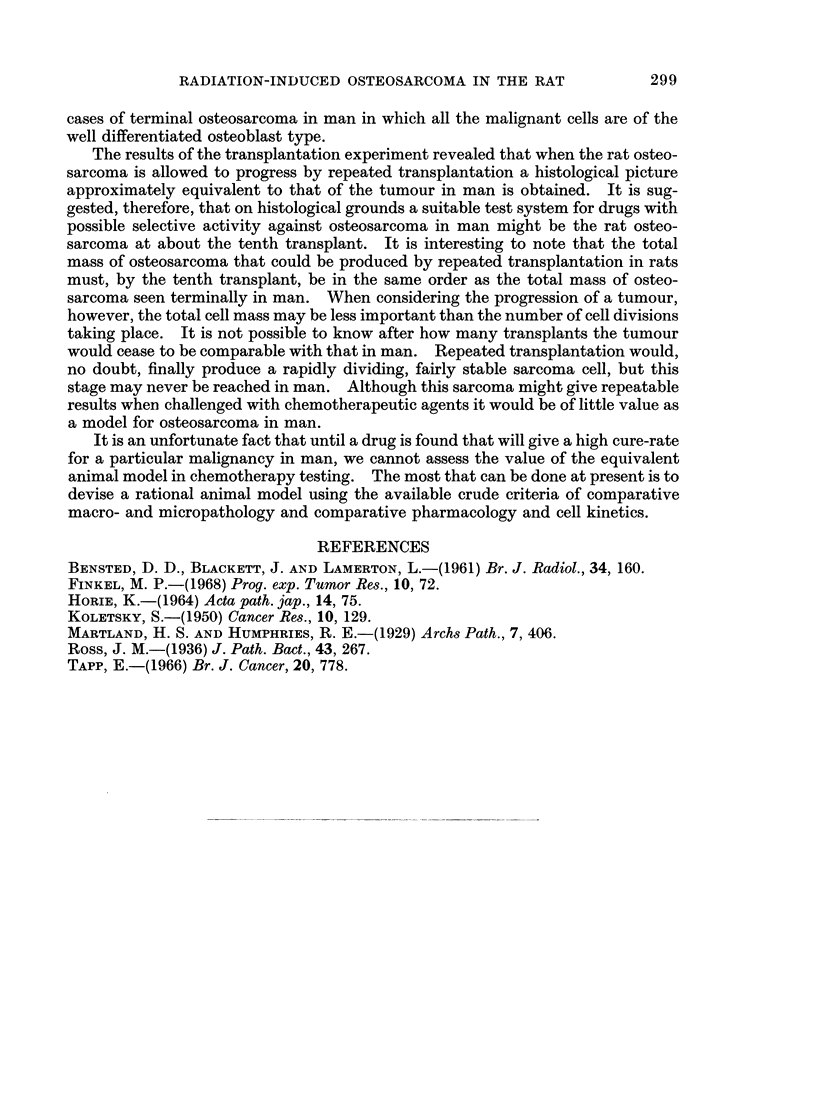# Radiation-induced Osteosarcoma in the Rat as a Model for Osteosarcoma in Man

**DOI:** 10.1038/bjc.1970.34

**Published:** 1970-06

**Authors:** L. M. Cobb

## Abstract

**Images:**


### RADIATION-INDUCED OSTEOSARCOMA IN THE RAT AS A MODEL FOR OSTEOSARCOMA IN MAN

L. M. COBB

*From the Chester Beatty Research Institute, Institute of Cancer Research, Royal Cancer Hospital, London, S.W.3*

Received for publication February 26, 1970

**SUMMARY.—A technique is described for the local induction of osteosarcoma in the rat by implanting $^{32}P$-impregnated polyvinyl chloride discs into the distal femoral metaphysis. The incidence of osteosarcomata after 18 months was 28%. A comparison is made of the pathology of the radiation-induced osteosarcoma in the rat and the " spontaneous " osteosarcoma in man. The possible value of the rat osteosarcoma model is discussed.**

THE purpose of the work described in this paper was to explore the possibility of using radiation-induced osteosarcoma in the rat as a model for osteosarcoma in man. The assessment of the value of tumours induced in laboratory animals as models for the study of malignancy in man has always been a thorny problem. Clearly, the value of the model will depend upon which aspect of malignant disease is under study. The particular interest of this laboratory is the chemotherapy of cancer. In order to study the response of the primary tumour in the animal to chemotherapeutic agents it would be of value to have a single accessible tumour. It was hoped to achieve this by implanting discs of $^{32}P$-impregnated polyvinyl chloride (PVC) into the distal metaphysis of the rat femur. This area was chosen because of its accessibility and the likelihood of a relatively high local osteoblastic activity.

The literature on the experimental induction of osteosarcomata in animals has recently been reviewed by Finkel (1968). Although the method of tumour induction used in the present work has not been described before, local implantation of radium (Ross, 1936) and beryllium (Tapp, 1966) have been used to produce osteosarcoma in the rabbit. When $^{32}P$ is injected parenterally in rats and mice osteosarcomata arise throughout the skeleton (Koletsky, 1950; Horie, 1964; Benstead, Blackett and Lamerton, 1961). In man we have the unfortunate history of radium-induced osteosarcoma in dial painters (Martland and Humphries, 1929). In addition, the occurrence of osteosarcoma following local radiotherapy is not always thought to be coincidental.

### MATERIALS AND METHODS

The source of radiation was $^{32}P$-impregnated sheets of PVC. The sheets are used clinically as a source of $\beta$-radiation for skin application (The Radiochemical Centre, Amersham, Bucks, U.K.). Two-mm. diameter discs were punched out of the 0·5 mm. thick sheet. Because it was not known if the phosphorus was uniformly distributed throughout the sheeting, 6 two-mm. discs were taken at random and applied to a diagnostic X-ray film. Seven days later the film was developed and six uniform zones of exposure indicated that $^{32}P$ distribution was adequately dispersed through the PVC.

Female rats were obtained from the Chester Beatty Research Institute random-bred colony of Wistar rats. In these animals the incidence of spontaneous osteosarcoma is negligible (Carter, 1969, personal communication). The discs were implanted under general anaesthesia when the animals were between 5 and 7 weeks of age. An incision was made over the lateral aspect of the knee joint and the protuberance of the lateral condyle identified. A two-mm. diameter trochar was used to bore a hole through the cortex of the femur one mm. proximal to the lateral protuberance (Fig. 1). The PVC disc was pushed through the hole into the medullary cavity where it could be expected to give maximum radiation to the metaphyseal area.

### EXPERIMENTS

### *Induction of osteosarcoma*

Group I. Single $^{32}P$-impregnated PVC discs were implanted into the distal femoral metaphysis of 100 rats. The PVC, at the time of implantation, had a surface dose rate in tissue-equivalent material of 27·6 rads/min. After 4 months the animals were examined twice weekly for evidence of osteosarcomata. When a swelling was felt in the distal femur weekly measurements were made of the suspected tumour. On no occasion did a suspected tumour subsequently regress. The animals were killed when the bulk of the tumour was impeding movement. A dorso-ventral view radiograph was taken of the whole animal under general anaesthesia immediately before death. All the remaining animals were killed 18 months after the implantation of the discs. This was 4 months after the appearance of the last osteosarcoma.

Group II. Single PVC discs were implanted into the distal femoral metaphysis of 100 rats. The same sheet of $^{32}P$-impregnated PVC was used for the discs of both groups I and II, but the discs for group II were not cut and used until the surface dose rate in tissue-equivalent material had fallen to 3·6 rads/min. After 4 months the animals were examined twice weekly for evidence of otseosarcoma. Those animals remaining 18 months after implantation were killed.

*Post mortem examination.*—When an animal died, or was killed, the following tissues were removed and fixed for histological examination: primary tumour, iliac and aortic lymph nodes, lung, heart, thyroid, liver, spleen, kidney, suprarenal, ovary and intestine. Gross pathology was recorded and sections prepared, and stained with haematoxylin and eosin for histological examination.

### *Transplantation experiment*

The lungs were removed under sterile conditions from one osteosarcoma-bearing rat. Small pieces of lung containing at least one visible metastasis were cut free and implanted into the peritoneal cavity of 6 female day-old rat littermates. The donor and recipient animals were only distantly related by random breeding. Between 16 and 29 days later tumour masses were observed in the abdomen of 5 of the 6 female recipients. Tumour from one of these females was transplanted subcutaneously into 6-week-old rats. This osteosarcoma has been transplanted as four strains (262, A, B, C, E) in female rats for more than 12

" generations ". Tissue was retained from the primary osteosarcoma and from all subsequent transplants of all four strains in order to observe the histopathological progression of the tumour.

### RESULTS

Osteosarcoma was diagnosed by histological examination in 26 rats in Group I (high radiation dose) and 1 rat in Group II (low radiation dose) (Table I). The diagnosis of osteosarcoma—malignant connective tissue tumour showing bone formation and thought to arise from osteoblasts—was made from material taken from the primary tumour arising at the irradiated site. Two rats with fibrosarcoma in the irradiated site occurred in Group I.

TABLE I.—*Tumour Incidence in Rats Following Local Irradiation*

| | Osteosarcomata | | | | Other tumours | |
|---|---|---|---|---|---|---|
| | Tumours appeared | Total | Incidence* | Number with lung metastases† | Fibrosarcoma | Mammary adenocarcinoma |
| Group I (100 rats) | 6–14 months | 26 | $\frac{26}{92}=28\%$ | 22 | 2 | 15‡ |
| Group II (100 rats) | 8 months | 1 | $\frac{1}{96}=1\%$ | 1 | 0 | 19‡ |

* The incidence of osteosarcoma was taken as a percentage of the animals still alive when the first osteosarcoma appeared, *i.e.* 6 months.

† Lung metastases were identified microscopically in the 22 rats, macroscopically in 12 and radiographically in 9.

‡ This is within the normal range for the incidence of mammary adenocarcinoma in the Chester Beatty colony.

### *Pathology of the osteosarcoma in the rat*

The osteosarcomata were identifiable by palpation at a diameter of 1·5–2 cm. At this stage the movement of the animal was not impeded, nor did it appear to be otherwise affected. The tumour gradually increased in size until at 4 to 8 weeks after the initial diagnosis, the tumour measured 5–7 cm. in diameter. At this stage movement of the animal was impeded and it was killed. On no occasion did

---

### EXPLANATION OF PLATES

FIG. 1.—*Implantation site.* The skin incision over the knee joint reveals two tendons and the lateral protuberance of the femur. The hole for the implantation of the PVC discs is made 1 mm. proximal to the protuberance.

FIG. 2.—*Primary osteosarcoma in the rat.* Well differentiated malignant osteoblasts forming bone. H. and E. × 300

FIG. 3.—*Cellular pleomorphism.* The eighth transplant of the rat osteosarcoma 262 E. Macronuclear cells and multinucleated cells emphasize the pleomorphism. There are scattered macrophages and polymorphonuclear leukocytes. H. and E. × 300

FIG. 4.—*Primary osteosarcoma.* Two weeks after physical recognition of a 2 cm. bony enlargement and 8 months after $^{32}P$ implantation. The X-ray plate shows osteosclerosis and early bone formation within the tumour mass. The triangular area of bone formation seen in the mid-shaft is produced by raised periosteum at the advancing edge of the tumour and is an important diagnostic pointer in man.

FIG. 5.—*Primary osteosarcoma.* X-ray plate 6 weeks after Fig. 4. The tumour mass has increased. In this animal lung metastases were clearly recognizable on X-ray plates.

FIG. 6.—*Primary fibrosarcoma.* A pseudarthrosis was noted 6 weeks before this tumour first became palpable. The pseudarthrosis is in the distal third of the femur. There is also a much more recent pathological fracture of the neck of femur.

1

2

3

4

Cobb.

5

6

a tumour, once identified, regress. Although lung metastases were frequently seen at *post mortem* (Table I) the rats were rarely dyspnoeic before they were killed. On those few occasions when there was dyspnoea the lung metastases were accompanied by patches of pneumonic change.

In order to compare the histopathology of the osteosarcoma in the rat with tumour in man, the results are given in tabulated form. It was only possible to generalize on the histopathological appearance of the tumours in the two species and examples could always be found which did not follow these generalizations. It should be noted that whereas in the rats the tumours were not treated in any way, osteosarcomata in man have usually had some form of therapy which might have altered the histopathological picture, although this tumour in man responds poorly to radiotherapy and is usually completely resistant to chemotherapy.

| RAT | MAN |
|---|---|
| The tumour is usually " gritty " to cut. Bone is formed more densely around the point of origin of the tumour than at the periphery. Although the muscle can be lifted away from the tumour there are distinct areas of infiltration of muscle by the tumour. The adjacent joint is infrequently involved. | The tumour is usually " gritty " to cut. Bone is formed more densely around the point of origin of the tumour than at the periphery. Although the muscle can be lifted away from the tumour there are distinct areas of infiltration of muscle by the tumour. The adjacent joint is infrequently involved. The tumour may have areas of haemorrhagic necrosis particularly after treatment. |
| The histology is mostly that of well differentiated malignant osteoblasts which form bone randomly, particularly in the older, central parts of the tumour (Fig. 2). Small areas of cartilage are seen in some tumours. Pleomorphism, spindle cell formation and giant cells are only rarely seen. Infiltration of muscle includes intrafibrillar spread of the tumour. | All the micropathologic changes seen in the rat can also appear in man but the bias is away from well differentiated osteoblasts and towards cellular pleomorphism. Giant cells with macronuclei, or multiple nuclei, are frequently present. Areas of spindle cells are also a common finding. |
| Metastatic spread from the primary tumour is most commonly to the lungs and infrequently to the regional lymph nodes. Microscopically the metastases resemble the primary tumour and retain its bone forming capacity. The lung metastases frequently spread along the pulmonary arterial tree. (Metastases were also found in the liver (2 rats), kidney (1 rat) and suprarenal (3 rats). | Metastatic spread from the primary tumour is most commonly to the lungs and infrequently to the regional lymph nodes. Microscopically the metastases resemble the primary tumour and retain its bone forming capacity. The lung metastases frequently spread along the pulmonary arterial tree. (Metastases also occur in the hilar lymph nodes, viscera and brain.) |

*Transplantation experiment*

A study was made of the histological progression in four strains of osteosarcoma, 262 A, B, C, E from the initial induced tumour to the tenth transplant generation. The initial osteosarcoma had extensive bone formation as did the lung metastases. The dominant cell was a well differentiated malignant osteoblast. With repeated transplantation the four strains developed the cellular pleomorphism commonly seen in man. There were multinucleated and macronuclear cells and areas of spindle cells (Fig. 3). Bone formation was still present in all four strains by the tenth transplantation but reduced in extent when compared with the initial tumour. (One of the strains (262 C) has subsequently developed a cystic degenerative change during the 12th and 13th transplantation and has " died out ".)

*Radiographic appearance of osteosarcoma in the rat*

The osteosarcomata were all clearly identifiable by osteosclerosis and bone formation (Fig. 4 and 5). Lung metastases could also be seen in 9 of the 12 animals which subsequently had visible lung metastases at *post mortem*. One of the 2 rats which developed fibrosarcoma had been observed to have a pseudarthrosis 4 months after implantation (Fig. 6). It seems most likely that the femur was damaged at the time of implantation and did not heal.

### DISCUSSION

The most suitable rat model for *early* osteosarcoma in man would probably be an osteosarcoma arising spontaneously in a random-bred colony. However, the natural incidence of this tumour in random-bred colonies is usually very low. As a substitute, osteosarcomata were induced in the present experiments by local radiation. It was difficult to estimate a suitable radiation dose. Too high a dose of local radiation might either produce necrosis and no tumours, or multiple local tumours—a situation which probably does not occur in man. While it might be expected that a low dose of radiation, giving an incidence of only one osteosarcoma in every 100 animals, would offer a more likely equivalent to the tumour in man, the cost of producing a sufficient number of tumours for a therapeutic trial would be prohibitive. It is suggested that the present incidence of 28% offers a reasonable compromise. It was of interest to note that the one osteosarcoma arising in the low radiation group (Group II), was indistinguishable radiologically and histologically from the majority of osteosarcomata in the higher radiation group (Group I). There is no way of knowing whether this one osteosarcoma was induced by the low local radiation and trauma or whether it arose spontaneously. It would be a purposeless exercise to attempt to assess the dosage received by osteoblasts in the area of the implant and so the manufacturer's specifications of surface dose rate in terms of tissue-equivalent material are given.

Although the induced rat osteosarcomata varied considerably in their histological appearance it was clear that the predominant cell was a malignant osteoblast and that cellular pleomorphism, which is a common feature in man, was rarely seen. One possible interpretation of this situation is that by the time osteosarcoma is diagnosed in man the tumour has already progressed beyond a " well differentiated osteoblast " stage. If this is so the induced osteosarcoma in the rat might be a suitable model for the study of pre-clinical early osteosarcoma in man. It would be of interest to review the histology of primary osteosarcomata in man in those rare cases where biopsy material was taken when the primary tumour was 1–2 cm. in diameter. Man may not, of course, ever pass through a " well differentiated osteoblast " stage. It should be noted that it is possible to find cases of terminal osteosarcoma in man in which all the malignant cells are of the well differentiated osteoblast type.

The results of the transplantation experiment revealed that when the rat osteosarcoma is allowed to progress by repeated transplantation a histological picture approximately equivalent to that of the tumour in man is obtained. It is suggested, therefore, that on histological grounds a suitable test system for drugs with possible selective activity against osteosarcoma in man might be the rat osteosarcoma at about the tenth transplant. It is interesting to note that the total mass of osteosarcoma that could be produced by repeated transplantation in rats must, by the tenth transplant, be in the same order as the total mass of osteosarcoma seen terminally in man. When considering the progression of a tumour, however, the total cell mass may be less important than the number of cell divisions taking place. It is not possible to know after how many transplants the tumour would cease to be comparable with that in man. Repeated transplantation would, no doubt, finally produce a rapidly dividing, fairly stable sarcoma cell, but this stage may never be reached in man. Although this sarcoma might give repeatable results when challenged with chemotherapeutic agents it would be of little value as a model for osteosarcoma in man.

It is an unfortunate fact that until a drug is found that will give a high cure-rate for a particular malignancy in man, we cannot assess the value of the equivalent animal model in chemotherapy testing. The most that can be done at present is to devise a rational animal model using the available crude criteria of comparative macro- and micropathology and comparative pharmacology and cell kinetics.

### REFERENCES

BENSTED, D. D., BLACKETT, J. AND LAMERTON, L.—(1961) *Br. J. Radiol.*, **34**, 160.
FINKEL, M. P.—(1968) *Prog. exp. Tumor Res.*, **10**, 72.
HORIE, K.—(1964) *Acta path. jap.*, **14**, 75.
KOLETSKY, S.—(1950) *Cancer Res.*, **10**, 129.
MARTLAND, H. S. AND HUMPHRIES, R. E.—(1929) *Archs Path.*, **7**, 406.
ROSS, J. M.—(1936) *J. Path. Bact.*, **43**, 267.
TAPP, E.—(1966) *Br. J. Cancer*, **20**, 778.